# Computational systems biology approaches for Parkinson’s disease

**DOI:** 10.1007/s00441-017-2734-5

**Published:** 2017-11-29

**Authors:** Enrico Glaab

**Affiliations:** Luxembourg Centre for Systems Biomedicine (LCSB), University of Luxembourg, 7 avenue des Hauts Fourneaux, L-4362 Esch-sur-Alzette, Luxembourg

**Keywords:** Parkinson’s disease, Systems biology, Pathway analysis, Network analysis, Bioinformatics

## Abstract

Parkinson’s disease (PD) is a prime example of a complex and heterogeneous disorder, characterized by multifaceted and varied motor- and non-motor symptoms and different possible interplays of genetic and environmental risk factors. While investigations of individual PD-causing mutations and risk factors in isolation are providing important insights to improve our understanding of the molecular mechanisms behind PD, there is a growing consensus that a more complete understanding of these mechanisms will require an integrative modeling of multifactorial disease-associated perturbations in molecular networks. Identifying and interpreting the combinatorial effects of multiple PD-associated molecular changes may pave the way towards an earlier and reliable diagnosis and more effective therapeutic interventions. This review provides an overview of computational systems biology approaches developed in recent years to study multifactorial molecular alterations in complex disorders, with a focus on PD research applications. Strengths and weaknesses of different cellular pathway and network analyses, and multivariate machine learning techniques for investigating PD-related omics data are discussed, and strategies proposed to exploit the synergies of multiple biological knowledge and data sources. A final outlook provides an overview of specific challenges and possible next steps for translating systems biology findings in PD to new omics-based diagnostic tools and targeted, drug-based therapeutic approaches.

## Introduction

Parkinson’s disease (PD) is one of the most common age-related, neurodegenerative disorders. In spite of 200 years of research on PD since its first published description by James Parkinson (Parkinson 1817), the disease etiology is still not fully understood. No disease-modifying therapy is available and no reliable diagnostic and progression biomarkers have so far been identified. The lack of a detailed molecular understanding and comprehensive mechanistic models for disease initiation and progression may at least in part be explained by the striking heterogeneity and complexity of the disease, which is manifested by a wide variety of motor and non-motor symptoms (Jankovic 2008; Solla et al. 2012; Müller et al. 2013; Kalia and Lang 2015). Recent genetic and epidemiological findings suggest that this high clinical heterogeneity is also reflected by a multitude of diverse PD risk factors and complex interplays between them (Gorell et al. 2004; Dardiotis et al. 2013; Kieburtz and Wunderle 2013). Known genetic influences include more than 20 loci associated with familial forms of PD and several risk factor variants identified for idiopathic PD (Kalinderi et al. 2016). Since about 15% of patients have a first-degree relative with PD (Samii et al. 2004) and only about 6–7% of an estimated total heritability of around 27% can be explained by the currently known PD-associated genetic variants (Do et al. 2011), several further genetic or epigenetic alterations may be involved in PD. This heritable component of the disease is complemented by multiple environmental risk factors implicated in PD etiology by epidemiological or Mendelian randomization studies, including exposure to toxic environmental agents, head injuries, and various drugs and dietary factors (Bellou et al. 2016). In analogy to the ‘dual-hit’ hypothesis previously proposed for other complex disorders (Knudson 1971), interplays of different factors may cause the disease and modulate the onset and severity of symptoms.

While studies on the influences of individual causal and risk-associated factors still represent an important information source, there is widespread agreement in the field that, in order to account for the ‘missing heritability’ in PD as well as the large proportion of idiopathic patients without a family history of PD, potential combinatorial effects of multiple genetic variations and/or environmental factors should be modeled and validated. Due to the large number of possible relevant molecular factors, an integrative modeling is not feasible using targeted experimental measurements and classical statistical methods alone, but additionally requires dedicated systems biology approaches, using high-throughput omics profiling techniques and bioinformatics approaches that exploit prior biological knowledge for data analysis.

This review presents a structured overview of current computational systems biology methods available for PD research, discusses their specific limitations and benefits, and highlights some of their recent applications in PD-related studies. First, methods for the analysis of PD-associated cellular pathway and molecular process alterations are compared, then related network analysis and causal reasoning approaches for identifying key regulatory factors are introduced. Next, machine learning approaches to build models for diagnostic sample classification and patient sub-group stratification are presented, including bioinformatics methods that exploit prior biological domain knowledge for integrative analyses. Because these approaches and their previous applications still suffer from several limitations, which have so far prevented the design of biomarker models for PD with sufficient accuracy, robustness and reproducibility, specific restrictions of prior work in this field are highlighted. As a final outlook, a discussion of PD-specific challenges and potential next steps for systems biology-based biomarker development and drug target identification is provided.

### Analyzing disease-associated activity changes in cellular pathways

A common first step towards understanding systems-level changes in omics datasets for complex diseases like PD is the investigation of molecular activity alterations in the context of known cellular pathways and molecular processes. For this purpose, a multitude of manually curated pathway and process definitions are available in public databases, including the Kyoto Encyclopedia of Genes and Genomes (KEGG; Ogata et al. 1999), the Gene Ontology database (GeneOntologyConsortium 2004), BioCarta (Nishimura 2001), WikiPathways (Pico et al. 2008), Reactome (Joshi-Tope et al. 2005) and the Pathway Interaction Database (Schaefer et al. 2009). Moreover, in addition to these generic pathway repositories, disease-specific resources have been established in recent years, providing dedicated pathway maps for the neurodegenerative disorders PD (see the PDMap; Fujita et al. 2014) and Alzheimer’s disease (see AlzPathway; Mizuno et al. 2012). When using a large and generic pathway database rather than a smaller selection of putatively relevant pathways for identifying disease-associated cellular process changes in an omics dataset, one has to consider that the final significance scores for an analysis will need to be adjusted for the number of tested hypotheses (equal to the number of pathways) to prevent excessive false positive discoveries (Benjamini and Hochberg 1995). Accordingly, using prior biological knowledge to pre-filter the considered pathways can be an effective strategy to increase the statistical power for showing significant associations.

Apart from selecting a pathway collection, researchers also need to choose between a wide range of statistical analysis approaches. In general, these omics-based pathway and geneset enrichment analysis methods can be grouped into four main categories (combining classifications previously proposed by Huang et al. 2009 and Di Lena et al. 2015):

1. Over-representation analysis (ORA): These approaches quantify the statistical over-representation of a list of genes, proteins or metabolites among the members of a pathway using a statistical test (e.g., Fisher’s exact test). The input list usually corresponds to the biomolecules which displayed a differential abundance in an omics dataset between a condition of interest (e.g., a disease state) as compared to a control condition, according to a chosen test statistic and significance threshold.
2. Geneset enrichment analysis (GSEA): GSEA methods avoid the need for defining a significance threshold and instead assign ranking scores to all biomolecules in the analyzed omics data to test whether the members of a pathway are ranked unexpectedly high or low among them (e.g., using modified versions of the Kolmogorov–Smirnov test).
3. Network module-based pathway analysis (NMPA): These algorithms exploit prior knowledge from molecular interaction networks to improve the scoring of pathway associations for omics profiling data. Typical NMPA methods first identify dense sub-network regions enriched in biomolecules undergoing activity changes (called “modules”), and, in a second step, quantify associations of these network modules with known pathways.
4. Network topology-based pathway analysis (NTPA): Similar to NMPA approaches, NTPA methods exploit molecular network information to obtain more robust and sensitive pathway association scores, but they avoid the initial module identification step and directly quantify pathway associations using graph-based statistics to assess the network distances and multiplicity of interconnections between the biomolecules of interest and pathway members mapped onto the network.

Table 1 shows an overview of representative, publicly available software tools for each of these four pathway analysis categories, including information on whether the tools are available as platform-independent web applications and whether they enable a visualization of the results.

**Table 1:** Publicly available software tools and web-applications for analyzing cellular pathway activity changes in omics datasets; some of the methods can be applied directly in the web browser (see column 4), and some of the tools provide advanced visualization features to facilitate the interpretation of the results (see column 5)

Method type	Software name	Availability	Web application	Visualization features	Reference
Over-representation analysis (ORA) tools	DAVID	https://david.ncifcrf.gov	Yes	No	Dennis et al. 2003
	GOstat	http://gostat.wehi.edu.au	Yes	Yes	Beißbarth and Speed 2004
	OntoExpress	http://vortex.cs.wayne.edu/ontoexpress	Yes	No	Draghici et al. 2003
	GoMiner	https://discover.nci.nih.gov/gominer	Yes	Yes	Zeeberg et al. 2003
	GOToolBox	http://genome.crg.es/GOToolBox	Yes	No	Martin et al. 2004
Geneset enrichment analysis (GSEA) tools	GSEA	http://software.broadinstitute.org/gsea	No	Yes	Subramanian et al. 2005
	GAGE	http://bioconductor.org/packages/release/bioc/html/gage.html	No	No	Luo et al. 2009
	GSA	http://statweb.stanford.edu/~tibs/GSA	No	No	Efron and Tibshirani 2007
	PAGE / PGSEA	https://www.bioconductor.org/packages/release/bioc/html/PGSEA.html	No	No	Kim and Volsky 2005
	GLOBALTEST	https://bioconductor.org/packages/release/bioc/html/globaltest.html	No	Yes	Goeman et al. 2004
	PADOG	http://bioconductor.org/packages/release/bioc/html/PADOG.html	No	No	Tarca et al. 2012
Network module-based pathway analysis (NMPA)	FunMOD	https://sourceforge.net/projects/funmodnetwork	No	Yes	Natale et al. 2014
	PINA	http://cbg.garvan.unsw.edu.au/pina	Yes	Yes	Cowley et al. 2012
	ReactomeFIViz	http://wiki.reactome.org/index.php/ReactomeFIViz	No	Yes	Wu et al. 2014
Network topology-based pathway analysis (NMPA)	PWEA	https://zlab.bu.edu/PWEA	No	Yes	Hung et al. 2010
	SPIA	http://bioconductor.org/packages/release/bioc/html/SPIA.html	No	Yes	Tarca et al. 2009
	PathNet	http://bioconductor.org/packages/release/bioc/html/PathNet.html	No	No	Dutta et al. 2012
	DeGraph	https://bioconductor.org/packages/release/bioc/html/DEGraph.html	No	Yes	Jacob et al. 2012
	EnrichNet	http://www.enrichnet.org	Yes	Yes	Glaab et al. 2012
	Ontologizer	http://ontologizer.de	Yes	Yes	Bauer et al. 2008
	SANTA	http://bioconductor.org/packages/release/bioc/html/SANTA.html	No	Yes	Cornish and Markowetz 2014
	ToPASeq	https://bioconductor.org/packages/release/bioc/html/ToPASeq.html	No	Yes	Ihnatova and Budinska 2015

When selecting a particular method among these choices, the following common limitations and benefits specific to different types of approaches should be considered: While the results of ORA methods are easy to calculate and interpret, they depend on the definition of a significance threshold and may not detect pathways enriched in many small molecular changes. By contrast, GSEA approaches do not require the specification of a significance cut-off and can identify pathways affected by strong cumulative effects of many small alterations. However, GSEA results are often difficult to interpret, and, as in ORA methods, the molecular network topology interconnecting the biomolecules of interest is not taken into consideration, since the statistics rely exclusively on available pathway annotations. This limitation is addressed by NMPA and NTPA approaches, which exploit information from gene regulatory, protein–protein or protein–metabolite interaction networks in order to increase the statistical power and robustness for identifying pathway-associated, co-ordinated network activity changes. Importantly, these software tools can account for the regulatory influences of biomolecules that have not yet been annotated for any known pathway. Moreover, they enable intuitive network visualizations, which can facilitate biological data interpretation. However, in contrast to pure network analysis methods (see the following section), NMPA and NTPA are hybrid approaches that combine aspects of both network and pathway analysis methods, and provide pathway rankings as the main output rather than altered sub-networks without known pathway annotations. As an important limitation, this also means that NMPA and NTPA approaches will not identify altered network regions that cannot be linked to any known cellular pathway. Moreover, a potential drawback in comparison to classical pathway analysis methods is that NMPA and NTPA statistics often rely heavily on the correctness and comprehensiveness of the underlying molecular interaction data. Similar to classical enrichment analyses, biases, noise, errors and incompleteness of the data used for network-based enrichment analyses can result in false-negative and false-positive findings. While sufficient high-quality molecular interaction data is typically available for the human species and common model organisms like mouse, rat, baker’s yeast and fruit fly, corresponding interaction data resources for other studied organisms may still be too incomplete for an effective application of these network analyses. Finally, similar to ORA approaches, NMPA and NTPA methods rely on differential expression thresholds, which need to be defined by the user.

The choice of a suitable pathway analysis approach is further complicated by the fact that many methods additionally require a prior computation of differential expression or abundance scores for the individual biomolecules in the studied omics data. This can be achieved using classical statistical approaches (e.g., the parametric *t* test or the non-parametric Mann–Whitney *U* test) or moderated statistical tests with improved feature variance estimation (Smyth 2004; Demissie et al. 2008), and by subsequently adjusting the *P* value significance scores for multiple hypothesis testing (Benjamini and Hochberg 1995). Discussions of these statistics for assessing changes in individual biomolecules and benchmark comparisons have been provided previously (Cui and Churchill 2003; Rapaport et al. 2013). Additionally, for pathway analyses of GWAS and sequencing datasets, specific technical issues have to be addressed, e.g., biases related to linkage disequilibrium, gene length and geneset size (see the discussion and guidelines by Wang et al. 2011 and Rahmatallah et al. 2015).

As a general recommendation for omics-based pathway analyses, it may often be helpful to compare at least a few of the above-mentioned alternative types of approaches, in order to identify different forms of biologically relevant alterations (e.g., pathways affected by few changes with large effect size/high significance, by many changes with small effect size/low significance, or by co-ordinated alterations in a specific sub-network of a pathway).

One of the first prominent examples for the application of pathway analysis approaches for PD research was a GSEA-based case-control study of post-mortem transcriptomics data from the midbrain (*substantia nigra*), using a weighted meta-analysis to combine effect size estimates for pathway-representing genesets across multiple independent datasets (Zheng et al. 2010). This analysis identified significant PD-associated alterations in 28 pathways, including 10 pathways subsequently validated in early subclinical cases of PD and in other PD-affected brain regions. Since the underexpression of a geneset of PGC-1α–responsive genes was significantly associated with PD pathology in this meta-analysis, the authors investigated PGC-1α over-expression as a new therapeutic strategy and reported that it suppressed dopaminergic neuron loss in two cell culture models of PD (Zheng et al. 2010).

A similar integrative pathway analysis study combined ORA statistics for PD-related GWAS and brain transcriptomics data to identify consensus pathway alterations across these two data modalities (Edwards et al. 2011). The authors used an unweighted meta-analysis approach (Fisher’s combined probability test) to integrate the significance scores for the genetic and gene expression data, and reported shared significant changes in multiple pathways, including the top three processes *axonal guidance*, *focal adhesion* and *calcium signaling*.

Apart from these studies focusing on human datasets, the integrated pathway-based analysis of data from PD-related animal models and human biospecimens has also been explored. By applying GSEA to 33 microarray datasets from human and animal model studies on PD, Oerton and Bender could show that the concordance across studies between summarized activity changes at the pathway-level was significantly higher than for individual differentially expressed genes (see fig. 2 in Oerton and Bender 2017). While only some animal model datasets revealed comparable changes to those in human studies, this study highlights that pathway analyses can help to address discrepancies between related omics studies at the level of single biomolecules due to technical and biological variance, and identify higher-level shared significant alteration signatures.

Finally, pathway analyses may also provide an effective means for cross-disease comparisons and for studying the molecular influences of factors associated with disease risk (e.g., aging, diet and toxin exposure). For example, an NTPA method revealed shared transcriptomics pathway alterations in the brain in PD and during adult aging (Glaab and Schneider 2015), and common inflammatory process changes in PD and Huntington’s disease were recently identified in a comparative pathway analysis of mRNA-seq data using a GSEA approach (Labadorf et al. 2017).

In summary, a wide choice of pathway analysis tools is available to study systems-level alterations in complex diseases, and their previous applications to PD-related omics data have already led to new insights on the processes affected by disease-related changes.

### Analyzing disease-associated molecular network alterations

While pathway-centric analyses can greatly facilitate the biological interpretation of omics data, the available public pathway definitions are often incomplete, may contain errors due to false-positive experimental discoveries, and inconsistencies can occur between subjectively defined boundaries for the same pathway across different databases (e.g., the “p53 signaling pathway” in KEGG differs significantly from the identically named pathway in the BioCarta database). As an alternative or extension to investigations based on pre-defined pathways, molecular network analyses have the potential to provide more detailed, comprehensive and novel findings for systems-level omics investigations. Network analyses do not require a time-consuming prior curation of cellular process annotations and avoid subjective judgments on the relevance of specific genes/proteins for a particular molecular function. They can exploit an extensive resource of interaction data from public databases, including STRING (Szklarczyk et al. 2015), BioGrid (Chatr-Aryamontri et al. 2015), IntAct (Kerrien et al. 2012), MINT (Licata et al. 2012), HPRD (Keshava Prasad et al. 2009) and HIPPIE (Schaefer et al. 2012), which cover significantly more biomolecular interactions than existing pathway databases.

However, a drawback of network analysis methods is that the results are often difficult to interpret; in particular, when the molecular changes of interest occur in a sub-network with few functionally annotated genes and no links to any known pathway. For this reason, hybrid approaches have been developed to combine the benefits of pathway and network analyses, e.g., algorithms to automatically extend existing pathway definitions via a graph-theoretic analysis of a surrounding genome-scale interaction network (Li et al. 2017). For network analyses in general, care must be taken to avoid biases: if data from small-scale protein interaction profiling studies is included in the network assembly, then frequently studied disease-related proteins may be biased to have larger numbers of identified interactors than other proteins. Therefore, either only data from genome-scale interaction profiling studies should be used or dedicated methods to reduce bias influences during the statistical sub-network analysis should be applied (e.g., see Ung et al. 2016).

Since a comprehensive discussion of biological network analysis approaches would extend beyond the scope of this review, only two of the most common method types are introduced here:

1. Network perturbation analyses (NPA): These methods aim to identify sub-networks within a genome-scale molecular or regulatory network that undergo co-ordinated activity changes in a biological condition of interest. Such co-ordinated network changes are characteristic for complex diseases, which tend to involve perturbations in the activity of entire molecular network regions rather than only in a few genes or proteins (Ideker and Sharan 2008; del Sol et al. 2010). NPA approaches typically consist of a search algorithm that heuristically explores the space of possible disease-affected sub-networks, and a scoring function that quantifies the overall significance and effect size of molecular changes in omics data mapped onto a sub-network. The final outcome of an NPA procedure is a ranking of the sub-networks with the most pronounced and robust alterations in the condition of interest as compared to a control condition.
2. Causal reasoning analyses (CRA): Causal reasoning (or causal network analysis) approaches use manually curated directional relationships, e.g., gene regulatory relationships or protein signaling cascades, to infer the root molecular causes for a set of observed condition-specific downstream changes in an omics dataset. While these directional relationships are often referred to as “causal relationships”, the underlying data are mostly correlational rather than causal and have to be interpreted with caution. By constructing a signed, directed interaction graph (often referred to as “causal graph” in the literature) from a list of known directional relationships between interacting molecules, a CRA method can track back through the graph from the molecules that underwent measured activity alterations in the omics data to their known or putative upstream regulators. These regulators are then scored as potential drivers of the observed downstream changes by evaluating the overall consistency of the activating and inhibitory regulation patterns in the graph with the measured data (see Chindelevitch et al. 2012). CRA studies enable the discovery of key regulatory molecules controlling specific biological processes of interest, e.g., a disease-related process.

**Fig. 1:**
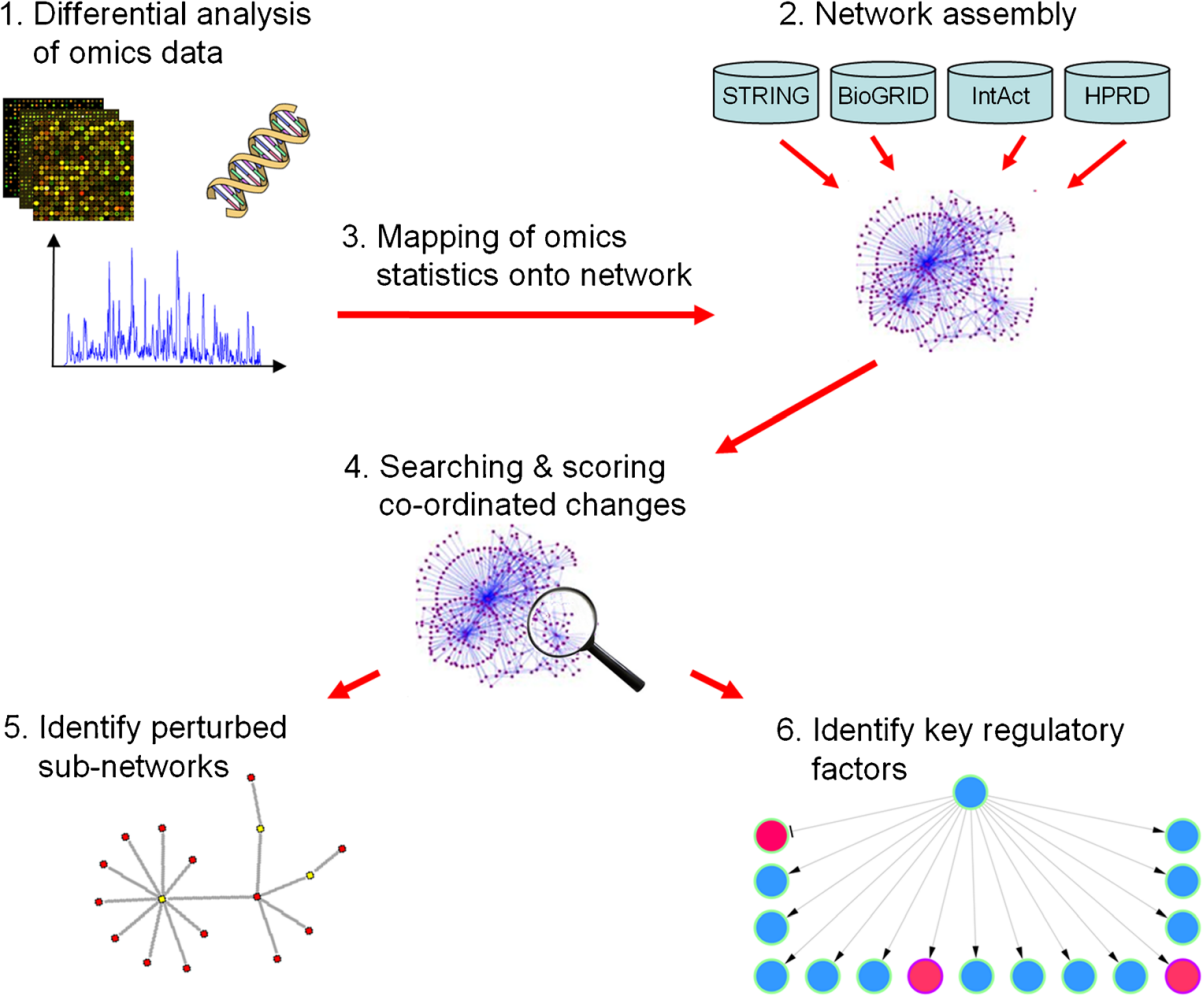
Overview of common steps in molecular network analyses of disease-related omics data

NPA and CRA methods are complementary methodologies with related purposes. NPA approaches help researchers to identify disease-associated co-ordinated activity changes across multiple biomolecules in a specific molecular network region, which may provide robust biomarker signatures for diagnostic applications. By contrast, CRA methods are mainly useful for identifying single upstream regulators with altered activity, which are responsible for a large fraction of observed downstream pathological changes and therefore of interest as potential drug targets for preclinical intervention studies. As a limitation, CRA software can only be applied to regulatory networks (represented by graphs with directed edges), whereas NPA tools are applicable to both regulatory and molecular interaction networks (represented by directed and undirected graphs). However, as illustrated in Fig. 1, for regulatory networks, the first steps of NPA and CRA approaches—consisting of the statistical omics data analysis, the network assembly and data mapping—are often identical or similar, so that NPA and CRA algorithms can be combined effectively within a single analysis pipeline. An overview of publicly available software tools for NPA and CRA is provided in Table 2, highlighting which of the tools provide network visualization features as opposed to a pure ranking functionality. Due to the high computational cost of most genome-scale network analyses, none of these tools are currently available as installation-free web applications; however, most of them can be installed on common desktop operating systems.

**Table 2:** Publicly available software tools for identifying sub-network perturbations and key regulatory biomolecules in omics datasets; some of the methods can be applied directly in the web-browser (see column 4), and some of the tools provide advanced visualization features to facilitate the interpretation of the results (see column 5)

Method type	Software name	Availability	Visualization features	Reference
Network perturbation analysis (NPA)	BioNet / HEINZ	http://www.bioconductor.org/packages/release/bioc/html/BioNet.html	Yes	Dittrich et al. 2008; Beisser et al. 2010, Dennis et al. 2003
	WMAXC	http://combio.gist.ac.kr/WMAXC/WMAXC.html	No	Amgalan and Lee 2014, Beißbarth and Speed 2004
	jActiveModules	http://apps.cytoscape.org/apps/jactivemodules	Yes	Ideker et al. 2002
	PinnacleZ	http://apps.cytoscape.org/apps/pinnaclez	Yes	Chuang et al. 2007
	COSINE	http://cran.r-project.org/web/packages/COSINE	Yes	Ma et al. 2011
	GenePEN	http://lcsb-portal.uni.lu/software/index.html	No	Vlassis and Glaab 2015
	MCWalk	https://bitbucket.org/akittas/biosubg	Yes	Kittas et al. 2016
	ClustEx	http://bioinfo.au.tsinghua.edu.cn/member/jgu/clustex	Yes	Gu et al. 2010
	BMRF	https://sourceforge.net/projects/bmrfcjava/	Yes	Chen et al. 2013
Causal reasoning analysis (CRA)	CRE	R source code available upon request from the author	Yes	Chindelevitch et al. 2012
	Whistle	https://github.com/Selventa/whistle	No	Catlett et al. 2013
	CausalR	https://bioconductor.org/packages/release/bioc/html/CausalR.html	Yes	Bradley and Barrett 2017
	QuaternaryProd	https://www.bioconductor.org/packages/release/bioc/html/QuaternaryProd.html	No	Fakhry et al. 2016
	BayesCRE	source code available upon request from the author	Yes	Zarringhalam et al. 2013
	MCWalk	https://bitbucket.org/akittas/biosubg	Yes	Kittas et al. 2016
	SigNet	https://cbdd.clarivate.com/cbdd	Yes	Jaeger et al. 2014

For network analyses in general, the result quality largely depends on how complete and correct the underlying network is, and corresponding methods may therefore not be applicable to model organisms with limited publicly available regulatory and interaction data. However, certain network analysis approaches, like the CRE method (see Table 2 and Chindelevitch et al. 2012), have been shown to be robust against considerable levels of noise in the input data.

In spite of the fact that many NPA and CRP algorithms have only been developed recently, multiple studies have already employed these approaches for PD molecular research. For example, Hu et al. (2017) manually curated the literature to define a set of 242 genes with previously reported genetic associations with PD, and investigated this geneset on a global human interactome using an NPA approach (the Steiner minimal tree algorithm, which is also implemented in the software *BioNet*; see Dittrich et al. 2008; Beisser et al. 2010). This resulted in the inference of a sub-network with PD-specific alterations, including new potential disease-related genes (Hu et al. 2017). In another study focusing on predictive network modeling and using transcriptomics data from the midbrain (*substantia nigra*) from PD cases and controls, the machine learning-based NPA approach, GenePEN, identified a connected sub-network signature within a genome-scale protein–protein interaction network with significant predictive power to distinguish between biospecimens from patients and unaffected subjects (Vlassis and Glaab 2015). As a further interesting application, PD-related brain transcriptomics data and an NPA method have been used to propose new functional links between microRNAs and PD, as well as new possible regulatory mechanisms for disease initiation and neuroprotection (Chandrasekaran and Bonchev 2013). Moreover, a first exemplary causal reasoning study subdivided differentially active pathways between brain transcriptomics samples from PD patients and controls into upstream and downstream processes, and ranked them hierarchically to propose new hypotheses on important upstream pathological alterations (Fu and Fu 2015). This approach suggested specifically that RNA metabolism pathology might be an upstream causal driver of PD pathogenesis.

Recently, network analysis techniques have also been employed as a means to compare PD to other complex diseases. Hypothesizing a relationship between PD and diabetes, Santiago and Potashkin (2013) mapped genes with known genome-wide significance in PD- and diabetes-related GWAS studies onto a human functional gene linkage network and identified a cluster of 478 genes closely associated with the seed genes for both diseases. Using a similar approach, Calderone et al. (2016) discovered shared and non-shared sub-networks associated with PD and Alzheimer’s disease, based on starting lists of genes derived from the public resources *PDMap* (Fujita et al. 2014) and *AlzPathway* (Mizuno et al. 2012). They then used functional and topological similarity measures to relate these sub-networks to biological processes in the Gene Ontology database, which pointed to associations with DNA repair, RNA metabolism and glucose metabolism, that could not be detected by a classical pathway enrichment analysis.

In summary, network perturbation and causal reasoning analyses are emerging as valuable complementary tools to conventional pathway analyses for the study of molecular changes in complex diseases. When significant pathological or protective activity changes occur in molecular sub-networks that still lack associated pathway annotations, only network analysis approaches are able to detect these alterations and predict new disease-associated processes and their main upstream regulators for subsequent experimental validation.

### Generating predictive machine learning models and visualizing high-dimensional data

One of the primary goals behind systems-level analyses of omics data for complex diseases is to identify biomarker signatures for differential diagnosis, patient sub-group stratification or disease prognosis. Generic machine learning software for diagnostic sample classification and clustering for patient sub-group stratification can often be applied ‘out-of-the-box’, without adaptations in the algorithms, to preprocessed omics data. However, in recent years, machine learning approaches that exploit prior biological domain knowledge as additional information source have been developed, which tend to provide more accurate, robust and biologically interpretable models than the classical generic methods (Fang et al. 2006; Lottaz et al. 2007).

Predictive model building typically starts with a feature selection or feature transformation step, eliminating uninformative attributes from the input omics data (e.g., by removing biomolecules with low activity variation across the samples) or combining the original attributes into more robust derived features (e.g., pathway-representing features, using weighted sums of measurements for pathway member biomolecules). These approaches are also called “dimension reduction methods”, because they reduce the number of dimensions of the input data (equal to the number of features) in order to address multiple common statistical issues during following analyses, previously summarized under the notion “curse of dimensionality” (Bellman 1961; Köppen 2000). Moreover, these methods enable the generation of low-dimensional visualizations of the data, e.g., 2D and 3D perspective plots, facilitating outlier detection and biological data interpretation.

**Fig. 2:**
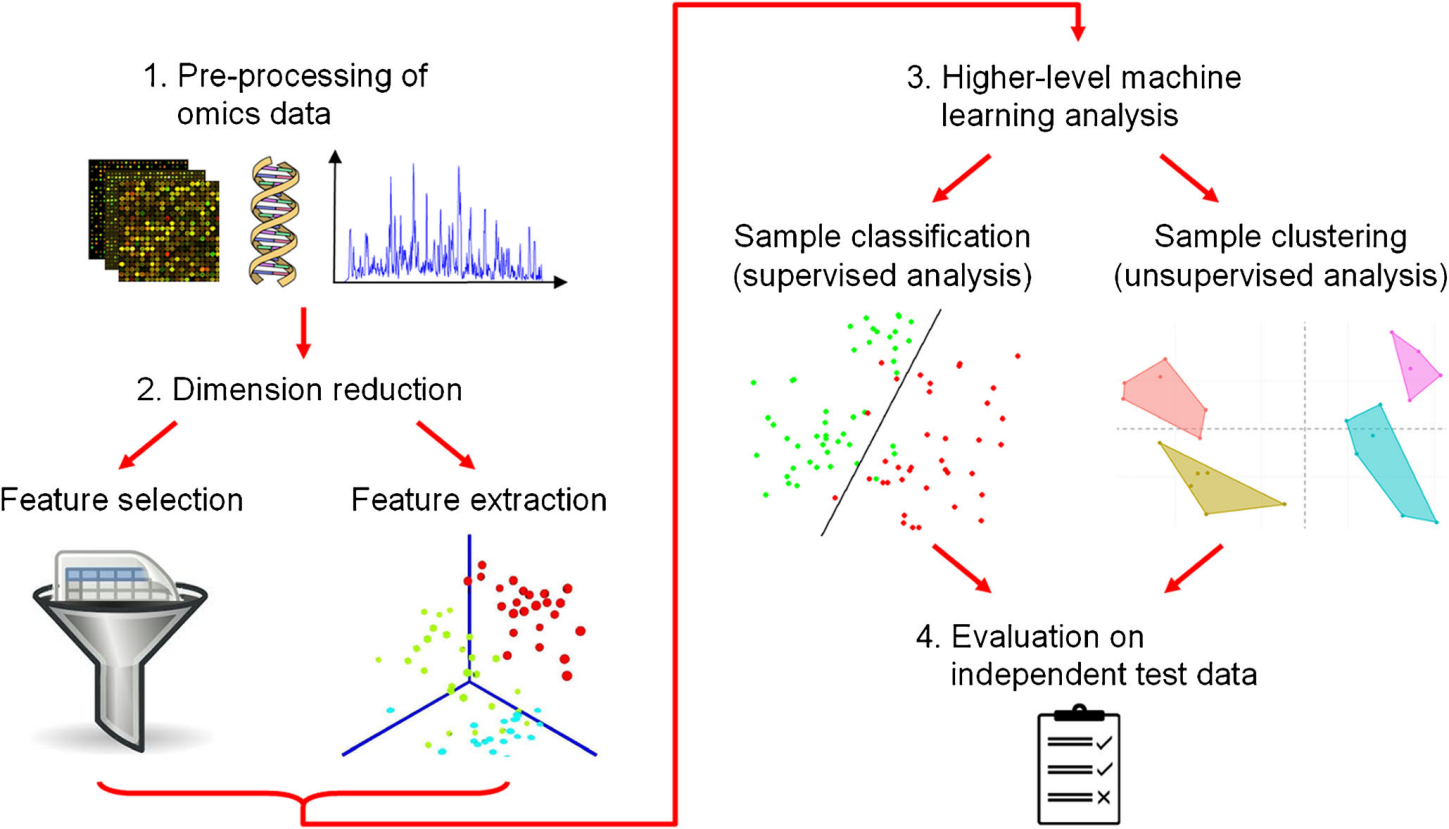
Common generic workflow for a machine learning analysis of omics data, including steps to reduce the dimension of the data through feature selection or feature extraction, higher-level machine learning analysis for classifying omics data samples (a supervised analysis) or clustering the samples (an unsupervised analysis), and evaluation of the obtained machine learning models on external test data

Table 3 provides an overview of dedicated software tools for machine learning analyses of omics data, including multi-purpose tool sets for sample clustering (unsupervised analysis) and classification (supervised analysis), software centered around the ranking and selection of informative attributes, and data visualization approaches (since a great variety of algorithms and implementations are publicly available, the table only highlights a representative selection with a focus on tools designed for systems biology data analysis). To illustrate how different types of methods can be interlinked within one analysis pipeline, Fig. 2 shows a common generic workflow.

**Table 3:** Overview of public software tools for predictive model building, clustering analysis and dimension reduction and visualization of omics data

Method type	Software name	Availability	Supported features^a^	Web application	Reference
Multi-purpose machine-learning analysis tool sets	CARMAWeb	https://carmaweb.genome.tugraz.at/carma/	N, P, C, D, V	Yes	Rainer et al. 2006
	ArrayMining	http://www.arraymining.net	N, P, C, D, V	Yes	Glaab et al. 2009
	mixOmics	https://cran.r-project.org/web/packages/mixOmics	P, D, V	No	Rohart et al. 2017
	Weka	http://www.cs.waikato.ac.nz/ml/weka	P, C, D, V	No	Hall et al. 2009
	Orange	https://orange.biolab.si	P, C, D, V	No	Demšar et al. 2013
	CMA	https://bioconductor.org/packages/release/bioc/html/CMA.html	P, D, V	No	Slawski et al. 2008
	MLSeq	https://bioconductor.org/packages/release/bioc/html/MLSeq.html	P, D	No	Zararsiz et al. 2014
Tools centered around feature ranking/feature selection	Limma	https://bioconductor.org/packages/release/bioc/html/limma.html	N, D, V	No	Smyth 2005
	RankProd	https://bioconductor.org/packages/release/bioc/html/RankProd.html	D, V	No	Hong et al. 2006
	ArrayPipe	http://www.pathogenomics.ca/arraypipe	N, D, V,	Yes	Hokamp et al. 2004
	RAP	https://bioinformatics.cineca.it/rap	N, D, V	Yes	D’Antonio et al. 2015
	EzArray	http://ezarray.com	N, D, V	Yes	Natale et al. 2014
Tools for low-dimensional data visualization	GGobi	http://www.ggobi.org	D, V	No	Temple Lang and Swayne 2001
	PlotViz	http://salsahpc.indiana.edu/plotviz	D, V	No	Choi et al. 2010
	RnavGraph	https://cran.r-project.org/web/packages/RnavGraph	V	No	Waddell and Oldford 2011
	Arena3D	http://arena3d.org	V	No	Secrier et al. 2012

^a^Column 3 highlights the supported features of the tools using the following codes:* N* normalization/preprocessing,* P* predictive model building,* C *unsupervised clustering,* D* dimension reduction (variable selection or feature transformation),* V* visualization. Tools available as web applications are highlighted in column 4

A major benefit of machine learning and visualization techniques for the analysis of omics data is their broad applicability. While different functional omics data types require different lower-level pre-processing methods, the higher-level machine learning and visualization tools discussed here are applicable across almost all types of pre-processed molecular data and often also support the integrative analysis of diverse omics types. Thus, given a pre-processed functional omics dataset, e.g., normalized microarray data, RNAseq read counts or mass-spectrometry-derived protein or metabolite abundance data, a wide variety of machine learning tools can be applied directly to identify clustering patterns (using unsupervised analyses) to build predictive models for the classification of new data samples (using supervised analyses), or to visually explore and interpret the data (using dimension reduction and visualization methods).

It is often recommendable to start the analysis of a normalized omics dataset with a simple visualization before using machine learning tools for automated pattern identification. Inspecting a 2D or 3D projection of the data can often reveal the presence of outliers, biases and other irregularities which are not always detected by automated quality control pipelines. The most commonly used approach for obtaining a low-dimensional visual representation of high-dimensional omics data is a principal component analysis (PCA). A benefit of PCA visualizations is that by design they tend to capture most of the variance in the data. However, in contrast to other dimension reduction methods like multidimensional scaling (MDS; Torgerson 1952), PCA is not designed to preserve original pairwise distances between data points when transforming the data to a low-dimensional space. Moreover, both PCA and MDS are linear approaches, which only tend to preserve distances between dissimilar data points in their low-dimensional representations, but in a linear data mapping it may often be impossible to keep highly similar data points close together (Van Der Maaten and Hinton 2008). Researchers may therefore want to consider some of the more recently developed non-linear data visualization approaches that focus on preserving local structure, e.g., locally linear embedding (Roweis and Saul 2000), Laplacian Eigenmaps (Belkin and Niyogi 2003) and t-SNE (Van Der Maaten and Hinton 2008).

After visual exploration of the omics data and the potential removal of outlier samples, the next steps for a machine learning analysis depend on the researcher’s specific goals and the availability of condition labels or outcome measures for the samples: if only unlabeled data with no related outcome measures are available, or if the analysis goal is to find distinct sub-groups among the samples (e.g., to stratify patients with distinct molecular alteration patterns), then unsupervised clustering approaches should be applied. These methods will identify sub-groups of samples that are similar to each other in terms of their omics profiles but differ significantly from other identified sub-groups. The relevant algorithms can be grouped into hierarchical clustering methods (e.g., hybrid hierarchical clustering, Chipman and Tibshirani 2006; Bayesian hierarchical clustering, Heller and Ghahramani 2005; and self-organizing maps, Ritter and Kohonen 1989), partition-based approaches (e.g., k-Means, Hartigan and Wong 1979; k-Mediods, Kaufman and Rousseeuw 1987; partitioning around mediods, Kaufman and Rousseeuw 1990) and density-based techniques (e.g., DBSCAN, Ester et al. 1996; DENCLUE, Hinneburg and Keim 1998; Chameleon, Karypis et al. 1999). While a detailed discussion of these methods and their biomedical applications extends beyond the scope of this review, a corresponding overview and guideline for algorithm selection has been provided previously (Andreopoulos et al. 2009).

A significant limitation of these generic clustering analyses of high-dimensional omics data is that, even after filtering the attributes by variance, many uninformative clustering patterns may still occur in the data. These are not necessarily spurious patterns, but may reflect real biological differences of the studied biospecimens (e.g., differences in gender, age, and dietary habits reflected by different biomolecular profiles) that could overshadow unrelated biomedically relevant differences between patient sub-groups of interest (e.g., disease sub-types with different treatment responses). Therefore, more recent approaches integrate prior biological knowledge into the cluster analysis, e.g., using gene/protein functional annotations and information from disease-related pathways, in order to aggregate measurements for functionally related, disease-associated biomolecules and determine more robust and relevant clustering patterns (Fang et al. 2006; Lottaz et al. 2007). For the subsequent evaluation of clustering results, no standard approach is available, but a variety of cluster validity indices have been proposed and should ideally be considered in combination (Kovács et al. 2005; Rendón et al. 2011; Arbelaitz et al. 2013). In general, ideal clusterings of patient biospecimens are characterized by low within-cluster distances and high between-cluster distances, are biologically interpretable and biomedically relevant, and replicable across different cohorts.

If class labels or quantitative outcome measures are available for the studied omics samples, reflecting biological conditions of interest (e.g., patient vs. control, or known disease sub-types) or measures of disease severity (e.g., scores from the Unified Parkinson’s Disease Rating Scale; Goetz 2003), then predictive models for diagnostic biospecimen classification can be built from the data by applying supervised machine learning approaches (relevant software tools are listed in Table 3 and highlighted by the code “P” for “prediction” in the third column). These algorithms use a set of omics data, called the “training set”, with known values for a chosen outcome variable, to identify patterns that enable a prediction of the outcome for new, unlabeled omics samples. By first applying a machine learning approach on the training set to generate a predictive mathematical function that relates patterns in the data to the outcome measure of interest, and then testing this predictive model on an independent set of omics samples with known outcomes (called the “test set”), the accuracy, sensitivity and specificity of the model can be estimated. More detailed guidelines on how to optimize machine-learning models using cross-validation and bootstrap procedures and how to evaluate the model performance on external tests set have already been provided elsewhere (Browne 2000; Braga-Neto and Dougherty 2004). Importantly, in particular when combining attribute selection methods with predictive machine learning, care must be taken to avoid selection biases (Wood et al. 2007).

In addition to classical generic statistical learning methods, new machine learning approaches guided by prior domain knowledge have been developed in recent years. These algorithms use dedicated multi-objective optimization approaches, which optimize the generated prediction models both by minimizing the training set error and by maximizing the consistency of the model with prior biological knowledge, or exploit biology-inspired data structures like ontology graphs or molecular networks for a structured data integration of multiple omics datasets. Representative examples for these types of approaches include the sparse overlapping Group Lasso approach for integrative multi-omics analysis (Park et al. 2015), which identifies driver genes in a biomedical omics datasets based on prior biological knowledge derived from predefined overlapping groups of features (e.g., gene functions in the Gene Ontology database), the network-constraint regularization approach for machine learning analysis of omics data by Li and Li (2008), and the multi-omics analysis approach by Mosca and Milanesi (2013), using a multi-objective optimization procedure to drive the identification of network regions enriched in molecular alterations across multiple omics data sources. A review by Li et al. (2016b) provides a more detailed overview on corresponding methods that exploit prior biological knowledge for integrative machine learning analysis of omics data.

In PD research, previous machine learning applications on omics data have mainly focused on diagnostic biomarker discovery in cerebrospinal fluid (CSF) and whole-blood samples. These efforts were motivated by the observation that even after the onset of visible motor symptoms, the currently used clinical diagnostic criteria for PD (UK Parkinson’s Disease Society Brain Bank criteria) only reached around 76% specificity in recent studies (increasing to 82% with retrospective application and 90% at death in a follow-up study; Berg et al. 2013). Importantly, while omics-based biomarker signatures for PD could in principle enable a more objective and accurate diagnosis, it is important to highlight that the previously proposed signatures have mostly not been reproduced or do not provide sufficient sensitivity and specificity for practical diagnostic purposes. This may largely be explained by limitations arising from small sample sizes, high biological and technical variance in the data, biases in the experimental procedures and instruments used for omics profiling (e.g., related to the machine, kit, experimenter, library or lane), no filtering of treatment/medication effects, no adjustment for common confounding factors, missing control samples for other neurodegenerative disorders, and the application of inadequate model building and validation techniques that result in over-fitted prediction models. Therefore, previous research on omics-based biomarker models for PD still represents preliminary work that has to be interpreted cautiously, and major technical and methodological challenges still have to be overcome to obtain clinically useful biomarker signatures.

A first representative metabolomics profiling study on blood samples from 66 PD patients and 25 unaffected controls reported a signature with 100% correct separation (Bogdanov et al. 2008). However, no cross-validation and no independent test set validation was performed, and, although some of the metabolite markers could be linked to known PD-associated processes, e.g., oxidative stress, the robustness and replicability of the prediction model has not yet been verified. Therefore, further study is needed, also in order to evaluate the extent to which the overall signature reflects PD-specific or generic disease-associated changes (e.g., blood inflammation-related markers are altered in many disorders).

Recently, a new metabolomics signature in CSF was proposed by Trezzi et al., using a non-targeted gas chromatography-mass spectrometry approach to study the CSF metabolome of 44 early-stage, untreated idiopathic PD patients in comparison with 43 age- and gender-matched unaffected controls (Trezzi et al. 2017). By applying a logistic regression approach, a machine learning model was trained to discriminate between patients and controls and tested on two independent validation sets (*n* = 18 and *n* = 38). The model involved the three metabolites mannose, threonic acid, and fructose as predictive features and was reported to provide a sensitivity of 0.79 and a specificity of 0.8. Additional studies including patients with other neurological disorders and larger numbers of samples from multiple cohorts are still needed to assess the predictive value of the signature for differential diagnosis.

Apart from metabolomics and proteomics signatures, gene expression changes in blood have also been considered as possible biomarkers for PD. Molochnikov et al. investigated gene transcription in blood samples from 62 early-stage PD patients and 64 unaffected controls and built a predictive model using stepwise multivariate logistic regression (Molochnikov et al. 2012). The resulting five-gene classification model was tested on an independent cohort of 30 advanced stage PD patients and 29 Alzheimer’s disease patients and separated them with 100% accuracy. However, no unaffected controls and no atypical forms of PD as disease control were included in the study validation set. Since Alzheimer’s disease is not associated with any motor symptoms similar to PD, assessing the potential of the proposed model for differential diagnosis of similar movement disorders will require additional investigations, including more disease conditions and larger sample sizes.

A further transcriptomics signature for PD was proposed by Scherzer et al., who used whole-blood microarray expression data from 105 subjects, covering 50 patients with early motor-stage PD, 33 control subjects with other neurological disorders and 22 unaffected controls (Scherzer et al. 2007). Their multigene marker was built by ranking and selecting genes in terms of their absolute Pearson correlation with binary sample class labels (representing PD vs. all controls), forming a template for each class from the mean values of the discriminating genes, and then defining a combined risk score for new biospecimen measurements corresponding to their Pearson correlation with the PD template minus its Pearson correlation with the non-PD template. The resulting signature was further validated in 39 independent test samples, but has so far not been replicated by independent research groups.

While most PD biomarker discovery approaches focus on data from idiopathic PD (IPD) patients, an interesting alternative approach using an integrative analysis of whole-blood gene expression data from IPD patients, familial PD patients with the *LRRK2* G2019S mutation and different mouse models was presented by Chikina et al. (2015). By first identifying differentially expressed genes between four groups of mice (overexpressing wild-type LRRK2, overexpressing G2019S LRRK2, LRRK2-knockout and wild-type mice) and combining them with previously proposed PD marker genes from the literature, a panel of 113 candidate marker genes was assembled and their expression measured for 34 symptomatic PD patients (both wild-type LRRK2 and G2019S LRRK2) and 32 asymptomatic controls using a digital gene expression platform. This led to the discovery of a subset of 14 markers discriminating between PD patients and asymptomatic controls with a reported accuracy of 79%. However, similar to other PD biomarker studies, no neurological disorder controls were included in the analysis, and further studies are required to determine whether the gene signature provides a significant informative value for differential diagnosis or whether it reflects a more generic inflammation response that may also occur in other disorders.

More recently, Shamir et al. presented a whole-blood gene expression signature for idiopathic PD, derived from microarray data analysis of 486 subjects (*n* = 205 PD, *n* = 233 controls, *n* = 48 other neurodegenerative diseases) (Shamir et al. 2017). Using batch-effect reduction and cross-validation procedures to prevent overfitting, their machine learning model included signatures of 100 genetic probes and was reported to reach a significant predictive performance on an independent validation cohort [area under the curve (AUC) = 0.79, *P* = 7.13E-6] and a subsequent independent test cohort (AUC = 0.74, *P* = 4.2E-4). The model was trained to differentiate between PD and unaffected controls rather than between PD and other neurologic disorders, and further analyses are needed to evaluate the potential of extending the model towards differential diagnostic applications, reducing the number of required genetic probes and increasing the generalization performance.

Multivariate machine learning methods have not only been applied for the analysis of PD-specific omics data but also for cross-disease comparisons between PD and other neurodegenerative disorders. In an exemplary study by Potashkin et al., splice variant-specific microarrays were used to find markers discriminating between whole-blood samples from 51 PD patients, 17 patients with multiple systems atrophy (MSA), 17 patients with progressive supranuclear palsy (PSP) and 39 unaffected controls (Potashkin et al. 2012). When applying a linear discriminant analysis to test the predictive accuracy of a signature of 13 selected differentially expressed, PD patients were reported to be distinguished from all controls with 96% sensitivity and 90% specificity and from the combined MSA and PSP patients with 94% sensitivity and 96% specificity. Seven of the 13 candidate markers were later confirmed to be dysregulated in PD on an independent set of whole-blood samples from 50 PD patients and 46 unaffected controls as part of a follow-up study by the authors (Santiago et al. 2013). While a major benefit of this work is that the baseline study considered two atypical forms or parkinsonism, MSA and PSP, in addition to PD, the signature has not yet been replicated by independent investigators and larger sample sizes for the neurodegenerative disorder controls will be required in future studies to evaluate the utility of the signature for differential diagnosis more precisely and robustly.

A further cross-disease comparative machine learning analysis presented by Abdi et al. (2006) involved a multiplex quantitative proteomics method, iTRAQ (isobaric tagging for relative and absolute protein quantification), applied in conjunction with multidimensional chromatography, followed by tandem mass spectrometry (MS/MS). This experimental procedure was used to compare the cerebrospinal fluid (CSF) proteome in patients with PD (*n* = 10), Alzheimer’s disease (*n* = 10), dementia with Lewy body (*n* = 5) and unaffected controls (*n* = 10). The authors determined a multifactorial marker signature using logistic regression, which was reported to provide a sensitivity of 78% and a specificity of 95% for discriminating between PD and the other disorders. Given the limited sample sizes in this study and the lack of an external replication, the authors acknowledge that their preliminary findings will have to be validated in a larger and independent population of patients.

Finally, a comparative machine learning analysis across multiple neurodegenerative disorders has also been performed by Ishigami et al., who used MALDI-TOF profiling of CSF peptides and proteins from 37 PD patients, 32 MSA patients and 26 control subjects with other neurological disorders (OND) (Ishigami et al. 2012). They applied a PCA for dimension reduction in combination with a support vector machine algorithm for supervised sample classification, and reported average cross-validated classification accuracies of 90.2% for distinguishing PD versus MSA and 98.2% for PD versus OND. The authors acknowledged that the sample size was small, and no independent replication has so far been conducted. Thus, additional external validation is still required to assess the generalization performance of this model.

Unsupervised machine learning approaches for patient sub-group identification in PD research have so far mainly been applied to clinical data. A first data-driven approach to characterize the heterogeneity in PD via clustering techniques was presented by Graham and Sagar (1999), who collected clinical information for 176 idiopathic patients and applied k-Means clustering to the normalized continuous variables. Their analysis suggested a separation of patients into three sub-groups at a disease duration of 5.6 years, and two sub-groups at 13.4 years. The identified sub-groups mainly differed in terms of measures of motor control and complications, age at onset and the degree of cognitive impairment. Similar studies by other research groups suggested a variety of different patient sub-groups: A two-group separation into rapid and slow progression (Gasparoli et al. 2002), a mild and a severely impaired group in terms of motor dysfunction and cognition (Dujardin et al. 2004), a young and an old onset group (Schrag et al. 2006), a three-group separation (Post et al. 2008), four alternative clusterings into four groups (Lewis et al. 2005; Reijnders et al. 2009; Liu et al. 2011; Van Rooden et al. 2011) and one five-group clustering (Lawton et al. 2015). The differences in the number and characteristics of the estimated clusters in these previous studies may mainly be explained by differences in the underlying patient cohorts and the considered features (e.g., only the study by Dujardin included SPECT measurements, and the clinical variables used across the studies differed significantly). While some of the proposed sub-types were reported to be reproduced in independent cohorts (Lewis et al. 2005; Reijnders et al. 2009; Van Rooden et al. 2011), in most studies no quantitative cluster validity index analyses were provided. Overall, further study is still warranted to derive and evaluate clinically relevant classification algorithms for PD patient sub-groups. A detailed review of stratification analysis results obtained so far, including recommendations on how to translate the gained knowledge into PD clinical research, has been provided by Marras and Lang (2013).

In summary, the previous application of machine learning methods for stratification and biomarker profiling analysis of PD suggest that multiple distinct sub-groups are present, and that significant disease-associated alterations occur in both CSF and blood. Given the limited sample sizes and restrictions in the types of neurodegenerative disorder controls available for biomarker profiling, further assessments are needed to determine whether the proposed signatures can be translated into clinically relevant tests for differential diagnosis with high robustness, sensitivity and specificity. Similarly, current stratification studies are partly hampered by restrictions in terms of the number and types of quantitative features considered, and in terms of the external statistical validation of clustering results. Future studies could address these limitations by combining further data types for robust cluster pattern identification, by assessing cluster correlations with independent clinically relevant variables and using additional quantitative external validations.

### Outlook on challenges and possible next steps for systems biology-based biomarker development and drug target discovery for PD

In recent years, the discovery of multiple PD-causing mutations and risk factor variants and the growth of public data resources for PD research, e.g., through the Parkinson’s Progression Markers Initiative (www.ppmi-info.org), have provided new means to pinpoint the main affected cellular pathways and gain a more detailed understanding of pathological changes in the disease. In order to translate the resulting knowledge and research efforts into improved diagnostic models and preclinical drug intervention studies, a variety of challenges still have to be overcome.

Since the midbrain (*substantia nigra*) is regarded as the main affected tissue in PD and only post-mortem omics data are available for this brain region, one of the main challenges for omics-based biomarker modeling is to identify reliable surrogate markers in peripheral tissues or body fluids. One possible strategy to address this in the future could be to use the non-lesional access to the brain during deep brain stimulation (DBS) surgery, by capturing cells spontaneously adhering to the DBS stylet for omics profiling (Zaccaria et al. 2016), and correlating these profiles to corresponding molecular measurements in blood samples. Using pathway and network analysis approaches discussed in this review, blood–brain correlations could not only be assessed at the level of individual biomolecules but also via pathway or sub-network activity scores to establish more robust correlations. A further strategy to explore could be the combined analysis of measurements for peripheral markers with limited specificity, e.g., biomarkers for oxidative stress in blood, with neuroimaging and clinical data using integrative machine learning methods. Classification models trained on individual data types could be combined via model averaging techniques (Dietterich 2000), or standardized features from the different data sources could be used to train a single, integrative prediction model. This synergistic modeling may help to address limitations of the individual data modalities and provide more robust and sensitive diagnostic models. Moreover, integrative analyses may reveal new interrelations across the different data types.

In order to obtain clinically relevant and reliable biomarker signatures for differential diagnosis, a further important objective for the future is to compare omics measurements for PD to sufficiently large sample sizes for atypical forms of PD and related neurodegenerative disorders. While it is challenging to recruit large numbers of atypical PD patients for a study, ongoing work on integrating information across different disease cohorts may help to address this issue.

A related hurdle in diagnostic model building is the general lack of statistical power in many studies. The high heterogeneity among PD patients, discussed earlier in this review, increases the variance in omics measurements and decreases the power to detect significant differences between patients and controls. Moreover, for PD, the number of publicly available omics data samples is much smaller as compared to Alzheimer’s disease and many cancer diseases. Larger sample sizes in combination with dimension reduction techniques and integrative analyses of multiple omics types will help to increase the power to identify new statistically significant PD-associated alterations and build more accurate prediction models. Moreover, computational approaches for combinatorial selection of biospecimens from a biobank for molecular profiling, designed to attain an optimal matching between patient and control samples in terms of multiple known confounding factors (age, gender, body-mass index, co-morbidities, smoking and dietary habits), provide a further possibility to increase the statistical power for comparative analyses at no added cost, but they are rarely used in practice. Bioinformatics methods can also facilitate biomarker discovery for the early pre-motor phase of PD, e.g., by combining analyses of molecular data from in vitro an in vivo models of early-stage PD with measurements from biospecimen of untreated de novo patients to pinpoint shared early-stage disease-associated changes. These integrative analyses could complement ongoing studies on the follow-up of at-risk cohorts, applying omics profiling analyses to biospecimens collected prior to the conversion to PD to discover presymptomatic molecular dysregulations.

For the specific goal of modeling and estimating the future progression of PD using omics data, time-series measurements from longitudinal studies will need to be collected in larger quantities and probably also at smaller time intervals. Current longitudinal studies for PD typically involve between one to two follow-up investigations per year. While important changes in the disease course may occur during shorter time periods, the burden for the patient through blood draws and clinical assessments needs to be minimized. Since many longitudinal studies so far only collect limited molecular data, a more comprehensive molecular phenotyping may currently deserve higher priority than narrowing the time interval between follow-up investigations. For the statistical analysis of time series data from corresponding studies, similar strategies to increase the statistical power can be applied as discussed for cross-sectional analyses in this review, e.g., using dimension reduction approaches, prior knowledge on interrelationships between biomolecules from pathways/networks and the literature, and integrative omics analyses to identify coordinated alteration trends over time. Representative examples of relevant time series analysis approaches for omics data have been presented by Wachter and Beißbarth (2014) and Lee et al. (2016).

Apart from the exploration of new omics measurements for biomarker modeling, the same data will also provide an important resource for systems biology analyses dedicated to the discovery, validation and characterization of PD drug targets. Network analyses including the causal reasoning approaches discussed in this review can help to identify pathological activity alterations in key regulatory proteins, and provide a starting point to prioritize candidate protein drug targets for further analyses. These investigations can be integrated with other more generic *in silico* target prioritization approaches (Aerts et al. 2006; Chen et al. 2009; Isik et al. 2015) and algorithms for scoring protein druggability via automated analyses of their molecular surface cavities in crystal structures (An et al. 2005; Volkamer et al. 2012). A limitation in the subsequent validation of pre-selected candidate targets using in vitro and in vivo disease models is that the current model systems for PD only reflect small subsets of the pathological features of PD as opposed to more established models for other complex disorders like Alzheimer’s disease (Beal 2001; Antony et al. 2011). Strategies involving the combined use of multiple complementary disease models, as well as ongoing projects on developing models with more robust pathological changes (e.g., using double knockouts of PD-mutated genes), will help to address these shortcomings. In this context, omics profiling and computational systems biology approaches will help to compare different disease models in terms of pathological and protective pathway activity changes and to assess their similarity to corresponding alterations in biospecimens from PD patients.

Finally, apart from their possible roles in the identification and preclinical validation of a drug target, systems biology approaches can also support the discovery of relevant drug-like small molecule binders. For example, a variety of systems-level approaches for drug repositioning have been developed (Dudley et al. 2011; Li and Lu 2013; Napolitano et al. 2013; Wu et al. 2013; Wang et al. 2014; Li et al. 2016a; Xu and Wang 2016), which can be complemented by virtual screening methods to identify new small molecule ligands for pre-selected targets (Stahura and Bajorath 2004; McInnes 2007). Further compound filtering is required due to the specific challenge for brain disorders that candidate drug-like molecules need to pass the blood–brain barrier (BBB). However, for compounds with unknown BBB permeability, dedicated *in silico* methods to predict this property are available as a prior filter for subsequent experimental testing (Kortagere et al. 2008; Muehlbacher et al. 2011; Carpenter et al. 2014). A more problematic common bottleneck is that extensive preclinical validation experiments for drug targets and their small-molecule binders are often not feasible in an academic setting in terms of the associated cost and resources, preventing promising target and compound discoveries from moving forward towards clinical development and testing. Projects that incentivize an earlier and more intensive collaboration between industry and academia on experimental target validation and preclinical drug development, e.g., the European Lead Factory (Mullard 2013), as well as the establishment of shared hardware and software infrastructures for systems biology (Athey et al. 2013; Auffray et al. 2016), will therefore be key facilitators for bridging the gap between new biomedical discoveries and their clinical translation.

In summary, computational systems biology approaches support experimental biomedical investigations by helping to prioritize candidate biomarkers, drug targets and binding compounds for subsequent validation, and providing insights into the mechanisms of molecular network and pathway dysregulations. For PD research specifically, integrative and comparative omics analyses that exploit prior biological knowledge can help to address current limitations in the available disease models and omics sample sizes, and to find surrogate markers for molecular changes in the brain. These computational systems-level analyses do not represent an alternative to targeted experimental studies of individual genes and proteins, but rather both targeted and systems-level approaches provide complementary information that will, collectively, help to pave the way towards improved biomarker signatures and new viable drug targets.
